# Retraction statement: RGB and HSV quantitative analysis of autofluorescence bronchoscopy used for characterization and identification of bronchopulmonary cancer

**DOI:** 10.1002/cam4.2961

**Published:** 2020-03-12

The above article from *Cancer Medicine*, published online on 5 October 2016 on Wiley Online Library (wileyonlinelibrary.com) and in Volume 5, pp. 3023–3030, has been retracted by agreement between the Authors, the journal Editor‐in‐Chief and John Wiley & Sons Ltd. The retraction has been agreed due to major overlap with a previously published article.

## References

[1] Zheng X , Xiong H , Li Y , Han B , Sun J . RGB and HSV quantitative analysis of autofluorescence bronchoscopy used for characterization and identification of bronchopulmonary cancer. Cancer Medicine. 2016;5(11): 3023–3030. 27709786 10.1002/cam4.831 PMC5119956

